# Unmasking Disparities in Gallbladder Cancer Outcomes in the Disaggregated Asian American Population

**DOI:** 10.1245/s10434-024-16168-x

**Published:** 2024-09-11

**Authors:** Keshav Kooragayala, Michael Wang, Francis J. Spitz, Tanay Veer Gandhi, John Dibato, Young Ki Hong

**Affiliations:** 1 Department of Surgery, Cooper University Hospital, Camden, NJ USA; 2 Cooper Medical School of Rowan University, Camden, NJ USA

## Abstract

**Background:**

Gallbladder cancer (GBC) is associated with a high mortality rate. Asian American (AsA) are among the fastest-growing populations in the United States, yet little is known about the disparity of GBC within this cohort. This study identified trends in treatment and outcomes for GBC in a disaggregated fashion, specifically for this population.

**Methods:**

A retrospective analysis of the National Cancer Database (NCDB) between 2010 and 2019 examining all patients treated for gallbladder cancer was performed. Basic demographic factors were identified for patients of Caucasian, African American, and disaggregated Asian subpopulations. Survival curves were used to identify differences in median overall survival, and a multivariate analysis was performed to determine which factors impact overall survival.

**Results:**

A total of 1317 (5%) patients were of AsA origin. Median survival for the overall AsA population is 15.1 months compared with Caucasian (11.5 months) and African Americans (11.4 months) (*p* < 0.0001). Within the AsA groups, the Korean subpopulation had the lowest survival at 12.6 months, whereas Filipinos had the longest survival at 19.1 months (*p* < 0.0001). Patients of Filipino descent had the highest rate of surgical resection but lower chemotherapy utilization. Conversely, Korean patients had the highest utilization of multimodality therapy. Multivariate analysis demonstrated that belonging to Chinese, Filipino, or Indian ethnicity was associated with decreased risk of mortality.

**Conclusions:**

There are disparate differences in survival for patients with GBC between AsA groups. Socioeconomic, genetic, and epigenetic factors may influence these differences. Further research is needed to delineate the causes of this disparity.

**Supplementary Information:**

The online version contains supplementary material available at 10.1245/s10434-024-16168-x.

Cancer is the leading cause of death in non-Hispanic Asian Americans and is the only ethnicity in which cancer mortality surpasses heart disease mortality.^1^ The Asian American (AsA) population represents approximately 7% of the U.S. population and is the fastest-growing ethnicity/race in the United States.^2^ Gallbladder cancer (GBC) is an aggressive cancer with a high mortality rate and poor prognosis owing to its asymptomatic nature of early disease. One study of the U.S. population showed that 85% of GBC cases were diagnosed after regional or distant spread to lymph nodes or organs had occurred.^3^ Estimates of 5-year survival of GBC after regional or distant metastasis are 28% and 3%, respectively.^4^ Worldwide, there are approximately 219,420 new cases of GBC annually, and in the United States, the rate of cases is 1.13 cases per 100,000 people each year.^3,5^

Most research studies aggregate the AsA population, which may mask treatment patterns and oncologic outcomes.^6^ This population is a heterogeneous group with considerable variability between specific ethnicities, especially regarding income, poverty rate, home ownership, and educational status.^7^ Recent studies have started to disaggregate this population in other cancers and have identified wide variations in outcomes.^8–12^ One paper demonstrated that Chinese, Korean, and Vietnamese patients had higher proportions of deaths from total cancers compared with other disaggregated AsA races.^8^ In a review looking at breast cancer, there were significant differences noted for screening, presentation, tumor biology, treatment, and survival between disaggregated Asian American races.^9^ Differences also were found in disaggregated analysis for pancreatic cancer, colon cancer, and prostate cancer.^10–12^ These studies all discuss the need for future literature to consider the Asian American population not as one homogenous comparison group, but as separate and unique patient populations.

This paper hypothesizes that patients with gallbladder cancer from different AsA subpopulations have differences in overall survival mediated by differences in cultural, socioeconomic, and epigenetic factors. The goal of this study was to identify the trends in treatment and outcomes for GBC in a disaggregated fashion, explicitly investigating the AsA population.

## Methods

### Cohort Selection

A retrospective analysis of the National Cancer Database (NCDB) between 2010 and 2019, examining all adult patients with gallbladder cancer, was performed. The methodology was derived from a recent study similarly assessing disaggregated outcomes for patients with pancreatic cancer.^10^ The NCDB is a joint project of the Commission on Cancer of the American College of Surgeons and the American Cancer Society, aggregating clinical data from hospital registries nationwide. The NCDB is estimated to include data from ~ 70% of all cancer cases diagnosed in the United States. The database included patients with clinical or pathologic stage I-IV gallbladder cancer. Asian American subpopulations were disaggregated and included Chinese, Japanese, Filipino, Korean, Vietnamese, and Indian populations. Although numerous other ethnic populations warrant recognition for their cultural heterogeneity, ethnic/racial groups that represented fewer than 1% of all cases were excluded. These populations were included in the “Other” cohort. Caucasian and African American patients were included as a reference population in our investigation, because they represent the ethnic subtypes with the most notable number of patients within the United States.

### Study Variables

Our primary outcome was median overall survival, stratified by race/ethnicity. Independent variables derived from the NCDB included race/ethnicity, age, gender, distance to treatment facility, income, Charlsen–Deyo comorbidity index, and insurance status.

### Statistical Analysis

Baseline characteristics were summarized separately by ethnicity, mean (SD), or median (first quartile, third quartile), as appropriate. Kaplan–Meier survival curves estimated the median overall survival for each ethnic group. Flexible parametric survival models were used to quantify the relative risk of overall deaths between ethnicities. These models were adjusted for age, sex, distance to treatment facility, facility type, insurance, income, treatments, clinical stage, pathologic stage, grade, and utilization of palliative care. The best-fitted model was chosen by using Akaike information criteria (AIC). Missing covariate data were included as a categorical field to retain the sample size. All analyses used SAS 9.4 (SAS Institute, Inc., Cary, NC) and R 4.2.2. Data were considered significant at *p* < 0.05.

## Results

**Fig. 1:**
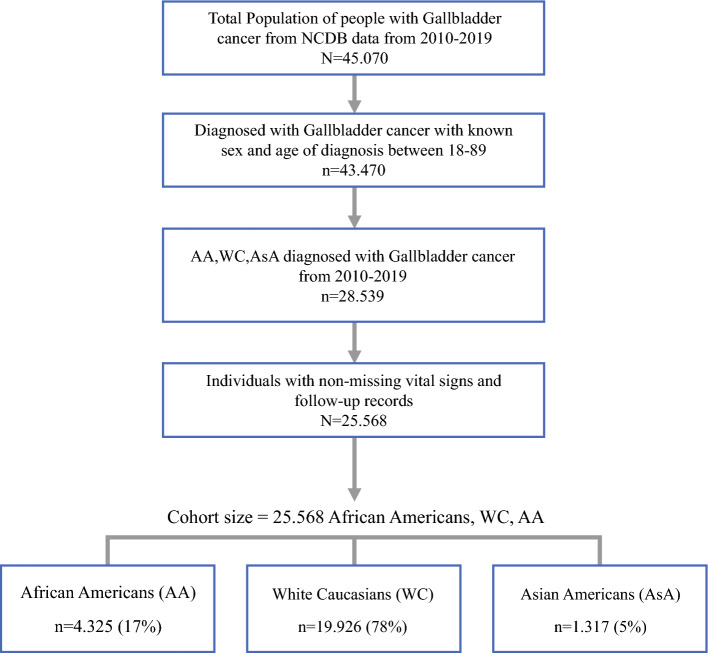
Selection of patients with gallbladder cancer from NCDB Data 2010–2019

There were 45,070 patients identified with GBC from 2010 to 2019 (Fig. 1). In this dataset, 1,317 patients (5% of the total) of AsA heritage were identified. Of these patients, Chinese patients accounted for 15.49% (*n* = 204), Japanese 6.23% (*n* = 82), Filipino 11.6% (*n* = 147), Korean 9.72% (*n* = 128), Vietnamese 7.44% (*n* = 98), Indian 20.50% (*n* = 270), and other 29.46% (388), which included Native Hawaiian and Pacific Islander ethnic groups. Of the total patients, 1440 (3%) were excluded because of missing data or unknown ethnicities. These groups comprised less than 2% of the total cohort and were excluded from the survival curves and multivariate analysis. A specific breakdown of each ethnic group is located in Supplementary Table 1. First, a comparison of baseline demographic data was performed between the individual AsA, Caucasian, and African American groups, representing most of the U.S. patients. In general, more patients from AsA heritage were treated at academic/research hospitals versus Caucasian patients but similar to African American patients (AsA: *n* = 575 [44%] vs. Caucasian: *n* = 6905 [35%] vs. African American *n* = 1925 [45%]). More AsA patients had private insurance than the other groups (AsA: *n* = 446 [34%] vs. Caucasian: *n* = 5,216 [26%] vs. African American: 1278 [*n* = 30]). A greater proportion of AsA patients were in the highest income bracket of >$63,333 compared with other groups (AsA: *n* = 625 [*n* = 47%] vs. Caucasian *n* = 6366 [32%] vs. African American: *n* = 757 [18%]). In terms of distance traveled to the treatment facility, Caucasian patients traveled the furthest (median distance 9.4 mi [interquartile range (IQR) 4.2–23.7] vs. AsA: 6.1 [IQR 3.3–12.1] vs. African American 6.6 [IQR 3.1–13.4]) (Table 1).

**Table 1:** Baseline demographics and treatment patterns for patients with GBC in NCDB between Caucasian, African-American, and AsA populations

	Overall		
	Caucasian	African-American	AsA
*N*	19,926	4325	1317
Follow-up, months	21.8 (27.0)	21.1 (25.9)	24.6 (28.5)
Age	69.6 (11.7)	66.2 (11.7)	67.0 (12.2)
Male (%)	6496 (33)	1304 (30)	477 (36)
Facility type			
Community cancer program	1458 (7)	228 (5)	79 (6)
Comprehensive community cancer program	7352 (37)	1241 (29)	411 (31)
Academic/research program	6905 (35)	1925 (45)	575 (44)
Integrated network cancer program	3974 (20)	864 (20)	224 (17)
Unknown	237 (1)	67 (2)	28 (2)
Insurance type			
Uninsured	701 (4)	193 (4)	47 (4)
Private	5216 (26)	1278 (30)	446 (34)
Government	13,709 (69)	2781 (64)	810 (62)
Unknown	300 (2)	73 (2)	14 (1)
Median income quartiles			
< $40,227	2806 (14)	1615 (37)	106 (8)
$40,227–$50,353	4023 (20)	809 (19)	164 (12)
$50,354–$63,332	4382 (22)	583 (13)	286 (22)
≥ $63,333	6366 (32)	757 (18)	625 (47)
Missing	2349 (12)	561 (13)	136 (10)
Distance from hospital, miles			
Nonmissing	17,770 (89)	3814 (88)	1182 (90)
Median (*q*1, *q*3)	9.4 (4.2, 23.7)	6.6 (3.1, 13.4)	6.1 (3.3, 12.1)
Urban rural			
Metro	16,389 (82)	3904 (90)	1261 (96)
Urban	2570 (13)	300 (7)	28 (2)
Rural	349 (2)	43 (1)	5 (0)
Missing	618 (3)	78 (2)	23 (2)
CDC score			
0	13,447 (67)	2717 (63)	919 (70)
1	4355 (22)	1054 (24)	291 (22)
2	1275 (6)	329 (8)	69 (5)
3	849 (4)	225 (5)	38 (3)
AJCC stage			
1	1121 (6)	295 (7)	83 (6)
2	2895 (15)	593 (14)	194 (15)
3	4614 (23)	937 (22)	319 (24)
4	8439 (42)	1922 (44)	543 (41)
Unknown	2857 (14)	578 (13)	178 (14)
Grade			
Well differentiated	1617 (8)	299 (7)	101 (8)
Moderately differentiated	4971 (25)	942 (22)	304 (23)
Poorly differentiated	4594 (23)	1101 (25)	291 (22)
Undifferentiated	284 (1)	55 (1)	23 (2)
Others	6080 (31)	1362 (31)	407 (31)
Cell type not determined	2380 (12)	566 (13)	191 (15)
Chemotherapy			
No	11750 (59)	2454 (57)	759 (58)
Yes	8176 (41)	1871 (43)	558 (42)
Surgery			
No	7122 (36)	1802 (42)	499 (38)
Yes	12,804 (64)	2523 (58)	818 (62)
Radiotherapy			
No	17,626 (88)	3791 (88)	1156 (88)
Yes	2300 (12)	534 (12)	161 (12)
Multiple treatments			
Chemotherapy + surgery + radiotherapy	1596 (8)	359 (8)	113 (9)
Chemotherapy + radiotherapy	329 (2)	87 (2)	21 (2)
Chemotherapy + surgery	3288 (17)	673 (16)	205 (16)
Chemotherapy only	2963 (15)	752 (17)	219 (17)
Surgery + radiotherapy	242 (1)	50 (1)	17 (1)
Radiotherapy only	133 (1)	38 (1)	10 (1)
Surgery only	7678 (39)	1441 (33)	483 (37)
None	3697 (19)	925 (21)	249 (19)
Surgical inpatient stay, days			
Nonmissing	11,199 (56)	2216 (51)	736 (56)
Mean (SD)	5.5 (10.2)	5.5 (8.4)	5.8 (9.7)
Facility location			
New England	1147 (6)	143 (3)	35 (3)
Middle Atlantic	3317 (17)	874 (20)	302 (23)
South Atlantic	3464 (17)	1443 (33)	164 (12)
East North Central	3591 (18)	707 (16)	103 (8)
East South Central	1067 (5)	298 (7)	12 (1)
West North Central	1638 (8)	141 (3)	50 (4)
West South Central	1824 (9)	434 (10)	67 (5)
Mountain	918 (5)	33 (1)	35 (3)
Pacific	2723 (14)	185 (4)	521 (40)
Unknown	237 (1)	67 (2)	28 (2)
Pathologic stage			
0	440 (2)	85 (2)	35 (3)
1	830 (4)	221 (5)	62 (5)
2	2315 (12)	466 (11)	153 (12)
3	3010 (15)	570 (13)	199 (15)
4	3848 (19)	832 (19)	226 (17)
Unknown	9483 (48)	2151 (50)	642 (49)
Palliative care	1978 (10)	452 (10)	135 (10)
Number of regional nodes	1.4 (3.6)	1.3 (3.3)	1.7 (4.1)
Regional nodes status			
Negative	3413 (17)	734 (17)	264 (20)
Positive	3161 (16)	623 (14)	243 (18)
Unknown	13,352 (67)	2,968 (69)	810 (62)

### Demographic Differences Among AsA Patients

The same analysis comparing demographic variables was performed between the individual AsA groups (Table 2). Generally, patients of Japanese origin were older than the average AsA patient (average age: 72.8 vs. 67.0 years). A considerable proportion of Japanese (77%), Filipino (67%), and Vietnamese patients (55%) lived on the Pacific coast. Conversely, more Indian patients lived in the mid-Atlantic region (39%). In addition, there were broad variations in facility utilization between ethnic groups; 51% of Chinese patients received treatment at academic/research facilities, followed by Indian (48%) and Korean (42%). More patients of Vietnamese origin (42%) and Filipino origin (35%) received care at Comprehensive Community Cancer Programs. There was wide variation in the distance patients traveled for their care. Chinese patients traveled the least distance at a median of 4.0 mi (IQR 2.1–7.9) compared with Korean (median distance: 7.2 mi [IQR 3.6–15.4]) or Filipino patients (median distance 7.2 mi [IQR 4.4–11.8]).

**Table 2:** Baseline demographics and treatment patterns of AsA patients with GBC in NCDB

	Chinese	Japanese	Filipino	Korean	Vietnamese	Indian	Others	Overall
N	204	82	147	128	98	270	388	1317
Follow-up, months	24.1 (29.8)	24.9 (27.4)	28.6 (29.8)	25.0 (30.4)	25.7 (31.4)	22.1 (26.3)	24.8 (27.7)	24.6 (28.5)
Age, years	68.8 (12.3)	72.8 (11.7)	68.2 (11.3)	69.7 (9.8)	68.2 (10.4)	63.6 (12.6)	65.4 (12.7)	67.0 (12.2)
Male (%)	85 (42)	27 (33)	42 (29)	55 (43)	39 (40)	90 (33)	139 (36)	477 (36)
Facility type								
Community cancer program	10 (5)	13 (16)	15 (10)	1 (1)	4 (4)	16 (6)	20 (5)	79 (6)
Comprehensive community cancer program	53 (26)	24 (29)	52 (35)	45 (35)	41 (42)	76 (28)	120 (31)	411 (31)
Academic/research program	104 (51)	25 (30)	56 (38)	54 (42)	33 (34)	130 (48)	173 (45)	575 (44)
Integrated network cancer program	36 (18)	19 (23)	22 (15)	28 (22)	19 (19)	37 (14)	63 (16)	224 (17)
Unknown	1 (0)	1 (1)	2 (1)	0 (0)	1 (1)	11 (4)	12 (3)	28 (2)
Insurance type								
Uninsured	4 (2)	0 (0)	3 (2)	6 (5)	2 (2)	13 (5)	19 (5)	47 (4)
Private	72 (35)	23 (28)	70 (48)	36 (28)	29 (30)	90 (33)	126 (32)	446 (34)
Government	126 (62)	59 (72)	74 (50)	86 (67)	67 (68)	163 (60)	235 (61)	810 (62)
Unknown	2 (1)	0 (0)	0 (0)	0 (0)	0 (0)	4 (1)	8 (2)	14 (1)
Median income quartiles								
< $40,227	24 (12)	2 (2)	5 (3)	4 (3)	8 (8)	19 (7)	44 (11)	106 (8)
$40,227–$50,353	31 (15)	12 (15)	14 (10)	9 (7)	17 (17)	21 (8)	60 (15)	164 (12)
$50,354–$63,332	37 (18)	16 (20)	27 (18)	26 (20)	24 (24)	76 (28)	80 (21)	286 (22)
≥ $63,333	96 (47)	40 (49)	90 (61)	80 (63)	31 (32)	129 (48)	159 (41)	625 (47)
Missing	16 (8)	12 (15)	11 (7)	9 (7)	18 (18)	25 (9)	45 (12)	136 (10)
Distance from hospital, miles								
Nonmissing	188 (92)	68 (83)	137 (93)	120 (94)	81 (83)	245 (91)	343 (88)	1,182 (90)
Median (*q*1, *q*3)	4.0 (2.1, 7.9)	4.8 (2.9, 10.9)	7.2 (4.4, 11.8)	7.2 (3.6, 15.4)	6.5 (3.9, 10.6)	7.1 (3.6, 12.1)	6.4 (3.6, 13.2)	6.1 (3.3, 12.1)
Urban rural								
Metro	200 (98)	74 (90)	139 (95)	124 (97)	94 (96)	260 (96)	370 (95)	1261 (96)
Urban	2 (1)	7 (9)	3 (2)	0 (0)	1 (1)	7 (3)	8 (2)	28 (2)
Rural	0 (0)	0 (0)	0 (0)	0 (0)	1 (1)	0 (0)	4 (1)	5 (0)
Missing	2 (1)	1 (1)	5 (3)	4 (3)	2 (2)	3 (1)	6 (2)	23 (2)
CDC score								
0	155 (76)	54 (66)	97 (66)	90 (70)	59 (60)	180 (67)	284 (73)	919 (70)
1	40 (20)	13 (16)	41 (28)	28 (22)	32 (33)	62 (23)	75 (19)	291 (22)
2	6 (3)	11 (13)	3 (2)	7 (5)	5 (5)	15 (6)	22 (6)	69 (5)
3	3 (1)	4 (5)	6 (4)	3 (2)	2 (2)	13 (5)	7 (2)	38 (3)
AJCC stage								
1	7 (3)	5 (6)	12 (8)	6 (5)	2 (2)	24 (9)	27 (7)	83 (6)
2	31 (15)	13 (16)	26 (18)	19 (15)	16 (16)	29 (11)	60 (15)	194 (15)
3	47 (23)	22 (27)	35 (24)	32 (25)	30 (31)	54 (20)	99 (26)	319 (24)
4	88 (43)	29 (35)	53 (36)	57 (45)	39 (40)	131 (49)	146 (38)	543 (41)
Unknown	31 (15)	13 (16)	21 (14)	14 (11)	11 (11)	32 (12)	56 (14)	178 (14)
Grade								
Well differentiated	16 (8)	8 (10)	10 (7)	8 (6)	7 (7)	24 (9)	28 (7)	101 (8)
Moderately differentiated	52 (25)	21 (26)	36 (24)	31 (24)	24 (24)	53 (20)	87 (22)	304 (23)
Poorly differentiated	38 (19)	20 (24)	31 (21)	34 (27)	25 (26)	64 (24)	79 (20)	291 (22)
Undifferentiated	2 (1)	1 (1)	4 (3)	2 (2)	5 (5)	4 (1)	5 (1)	23 (2)
Others	68 (33)	21 (26)	52 (35)	42 (33)	25 (26)	81 (30)	118 (30)	407 (31)
Cell type not determined	28 (14)	11 (13)	14 (10)	11 (9)	12 (12)	44 (16)	71 (18)	191 (15)
Chemotherapy								
No	120 (59)	48 (59)	99 (67)	72 (56)	57 (58)	140 (52)	223 (57)	759 (58)
Yes	84 (41)	34 (41)	48 (33)	56 (44)	41 (42)	130 (48)	165 (43)	558 (42)
Surgery								
No	92 (45)	29 (35)	48 (33)	51 (40)	35 (36)	106 (39)	138 (36)	499 (38)
Yes	112 (55)	53 (65)	99 (67)	77 (60)	63 (64)	164 (61)	250 (64)	818 (62)
Radiotherapy								
No	183 (90)	63 (77)	126 (86)	116 (91)	84 (86)	238 (88)	346 (89)	1156 (88)
Yes	21 (10)	19 (23)	21 (14)	12 (9)	14 (14)	32 (12)	42 (11)	161 (12)
Multiple treatments								
Chemotherapy + surgery + radiotherapy	15 (7)	11 (13)	14 (10)	8 (6)	10 (10)	25 (9)	30 (8)	113 (9)
Chemotherapy + radiotherapy	4 (2)	4 (5)	2 (1)	0 (0)	2 (2)	2 (1)	7 (2)	21 (2)
Chemotherapy + surgery	28 (14)	7 (9)	11 (7)	25 (20)	13 (13)	47 (17)	74 (19)	205 (16)
Chemotherapy only	37 (18)	12 (15)	21 (14)	23 (18)	16 (16)	56 (21)	54 (14)	219 (17)
Surgery + radiotherapy	2 (1)	4 (5)	4 (3)	1 (1)	1 (1)	3 (1)	2 (1)	17 (1)
Radiotherapy only	0 (0)	0 (0)	1 (1)	3 (2)	1 (1)	2 (1)	3 (1)	10 (1)
Surgery only	67 (33)	31 (38)	70 (48)	43 (34)	39 (40)	89 (33)	144 (37)	483 (37)
None	51 (25)	13 (16)	24 (16)	25 (20)	16 (16)	46 (17)	74 (19)	249 (19)
Surgical inpatient stay, days								
Nonmissing	107 (52)	44 (54)	95 (65)	70 (55)	59 (60)	145 (54)	216 (56)	736 (56)
Mean (SD)	4.7 (7.2)	5.0 (4.9)	6.7 (17.1)	5.2 (5.2)	8.1 (9.5)	5.5 (9.6)	5.8 (8.2)	5.8 (9.7)
Facility location								
New England	6 (3)	0 (0)	1 (1)	1 (1)	1 (1)	6 (2)	20 (5)	35 (3)
Middle Atlantic	71 (35)	5 (6)	22 (15)	28 (22)	5 (5)	106 (39)	65 (17)	302 (23)
South Atlantic	18 (9)	6 (7)	12 (8)	24 (19)	10 (10)	43 (16)	51 (13)	164 (12)
East North Central	7 (3)	2 (2)	3 (2)	7 (5)	5 (5)	30 (11)	49 (13)	103 (8)
East South Central	0 (0)	0 (0)	0 (0)	1 (1)	0 (0)	5 (2)	6 (2)	12 (1)
West North Central	2 (1)	2 (2)	2 (1)	3 (2)	3 (3)	9 (3)	29 (7)	50 (4)
West South Central	7 (3)	1 (1)	1 (1)	3 (2)	14 (14)	13 (5)	28 (7)	67 (5)
Mountain	2 (1)	2 (2)	5 (3)	3 (2)	5 (5)	9 (3)	9 (2)	35 (3)
Pacific	90 (44)	63 (77)	99 (67)	58 (45)	54 (55)	38 (14)	119 (31)	521 (40)
Unknown	1 (0)	1 (1)	2 (1)	0 (0)	1 (1)	11 (4)	12 (3)	28 (2)
Pathologic stage								
0	5 (2)	1 (1)	8 (5)	2 (2)	2 (2)	9 (3)	8 (2)	35 (3)
1	6 (3)	4 (5)	9 (6)	5 (4)	2 (2)	14 (5)	22 (6)	62 (5)
2	22 (11)	11 (13)	22 (15)	17 (13)	13 (13)	24 (9)	44 (11)	153 (12)
3	32 (16)	12 (15)	20 (14)	21 (16)	21 (21)	29 (11)	64 (16)	199 (15)
4	39 (19)	10 (12)	26 (18)	18 (14)	19 (19)	62 (23)	52 (13)	226 (17)
Unknown	100 (49)	44 (54)	62 (42)	65 (51)	41 (42)	132 (49)	198 (51)	642 (49)
Palliative care	26 (13)	9 (11)	14 (10)	15 (12)	11 (11)	28 (10)	32 (8)	135 (10)
No. regional nodes	1.4 (3.2)	1.9 (5.1)	1.6 (3.0)	1.3 (2.8)	1.4 (2.5)	1.9 (4.2)	2.0 (5.2)	1.7 (4.1)
Regional nodes status								
Negative	35 (17)	11 (13)	41 (28)	22 (17)	15 (15)	52 (19)	88 (23)	264 (20)
Positive	32 (16)	14 (17)	20 (14)	24 (19)	26 (27)	53 (20)	74 (19)	243 (18)
Unknown	137 (67)	57 (70)	86 (59)	82 (64)	57 (58)	165 (61)	226 (58)	810 (62)

### Differences in Survival

**Fig. 2:**
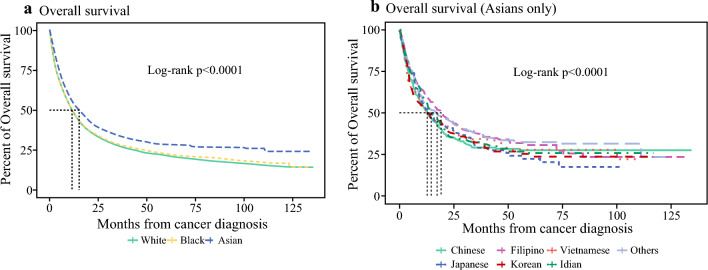
Overall survival of Asian American population with gallbladder cancer in the NCDB cohort

**Fig. 3:**
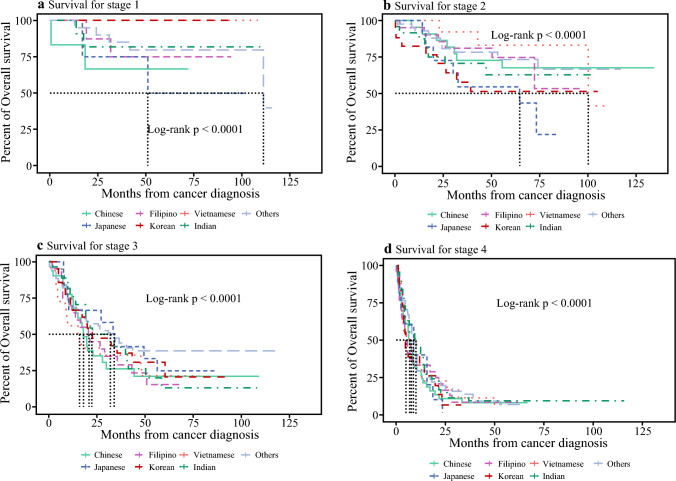
Variable OS between individual AsA cohorts stratified by stage of disease

Median overall survival (OS) was highest for the AsA group compared with Caucasian and African American cohorts within the United States (AsA 15.1 months [IQR 13–17.3] vs. Caucasian 11.5 months [IQR 11.2–11.8] vs. African American 11.4 months [IQR 10.6–12.1], *p* < 0.0001) (Fig. 2). Within the individual AsA, there were noteworthy variations in survival between groups. Filipino patients had the highest overall survival (19.1 months [IQR 13.96–28.3]), followed by Indian (14.6 months [IQR 12.52–19.1]) and Japanese patients (14.1 months [IQR 9.43–27.2]) (Fig. 3). The lowest median survival was found amongst Vietnamese patients (12.7 months [IQR 9.13–25.8]). Although limited with the sample size to generate survival curves for patients with Stage 1 and 2 disease, a survival curve for Stage 3 disease could be generated. This curve showed that Japanese patients had the highest OS (33.9 months [IQR 10.4–NA]), whereas Vietnamese patients had the lowest OS (16 months [IQR 8.9–NA]).

### Differences in Disease Characteristics and Treatment

Of all patients within the AsA cohort, Filipino patients were diagnosed with the least number of patients with clinical stage 4 disease (36%), whereas 49% of Indian patients had the highest (Table 2). Treatment utilization also varied between each cohort. Surgical resection, which is a mainstay of therapy for nonmetastatic disease, was utilized by 67% of Filipino patients, followed by Japanese (65%) and Vietnamese (64%) patients. Chemotherapy was used the most by Indian patients (48%) and least by Filipino patients (33%). While many patients received multimodality treatment, 19% of all AsA patients did not receive any cancer-directed therapy. Within individual cohorts, 25% of Chinese patients received no treatment. Of note, 47% of Filipino patients only underwent surgery with no adjuvant or neoadjuvant therapy compared with the average of 37% of all AsA groups. There was minimal variation in the utilization of palliative care between groups.

### Factors Associated with Increased Mortality

After factoring in covariates in our multivariate analysis, Chinese, Indian, and Filipino descent was associated with decreased mortality (Table 3). Conversely, older age, increasing Charlson Dayo score, treatment at facilities other than academic/research centers, and growing stage of disease were all associated with increased mortality.

**Table 3:** Multivariate analysis of factors associated with increased overall mortality for GBC

	Whole cohort
Race	
White	Ref
Black	0.91 (0.87, 0.94)
Chinese	0.79 (0.66, 0.94)
Japanese	0.94 (0.72, 1.21)
Filipino	0.73 (0.59, 0.88)
Korean	0.84 (0.67, 1.03)
Vietnamese	0.86 (0.67, 1.09)
Indian	0.86 (0.73, 0.99)
Others	0.79 (0.69, 0.89)
Age	
< 60	Ref
60+	1.21 (1.19, 1.23)
Urban rural	
Metro	Ref
Urban	1.09 (1.05, 1.14)
Rural	0.99 (0.88, 1.11)
Sex	
Male	Ref
Female	0.96 (0.94, 0.98)
CDC score	
0	Ref
1	1.08 (1.05, 1.12)
2	1.19 (1.12, 1.26)
3	1.29 (1.21, 1.38)
Hispanic	
No	Ref
Yes	0.76 (0.73, 0.81)
Facility type	
Community cancer program	Ref
Comprehensive community cancer program	0.98 (0.96, 1.01)
Academic/research program	0.83 (0.81, 0.85)
Integrated network cancer program	1.01 (0.97, 1.04)
Insurance type	
Uninsured	Ref
Private	1.01 (0.98, 1.04)
Government	1.13 (1.11, 1.15)
Median income quartiles	
< $40,227	Ref
$40,227–$50,353	1.01 (0.97, 1.04)
$50,354–$63,332	1.0 (0.97, 1.04)
≥ $63,333	0.94 (0.92, 0.96)
Grade	
Well differentiated	Ref
Moderately differentiated	1.47 (1.43, 1.52)
Poorly differentiated	2.03 (1.97, 2.09)
Undifferentiated	2.08 (1.84, 2.33)
Multiple treatments	
None	Ref
Chemotherapy + surgery + radiotherapy	0.15 (0.14, 0.16)
Chemotherapy + radiotherapy	0.32 (0.28, 0.36)
Chemotherapy + surgery	0.21 (0.20, 0.22)
Chemotherapy only	0.40 (0.38, 0.41)
Surgery + radiotherapy	0.21 (0.18, 0.24)
Radiotherapy only	0.48 (0.40, 0.57)
Surgery only	0.27 (0.26, 0.28)
Pathologic stage	
0	Ref
1	1.60 (1.45, 1.76)
2	2.59 (2.47, 2.73)
3	5.37 (5.17, 5.57)
4	7.89 (7.65, 8.13)
Palliative care	1.36 (1.30, 1.42)
No. regional nodes	0.97 (0.96, 0.97)

Data are hazard ratios with 95% confidence intervals in parentheses

## Discussion

There are approximately 22 million Asian Americans currently in the United States, comprising approximately 7% of the population.^7^ This highly heterogeneous demographic from more than 20 countries is the fastest-growing demographic in the United States, projected to surpass 46 million people by 2060. Gallbladder cancer is an aggressive form of cancer with poor outcomes. The current relative 5-year survival rate for Asian Americans with gallbladder cancer is 22.1%, which is slightly higher than the U.S. relative 5-year survival rate of 20.9%.^13^ Gallbladder cancer often starts with chronic inflammation of the area, which then disrupts normal cell growth. Major factors for gallbladder cancer include age, obesity, genetics, occupational exposure to mutagens, chronic infection, and gallstones. Chronic infections with *Salmonella* (e.g., *S. typhi* and *S. paratyphi*) or *Helicobacter* (*H. pylori* and *H. bilis*) have been associated with gallbladder cancer. *Salmonella enterica* serovar Typhi, which causes typhoid fever, also has been associated with gallbladder cancer. Areas where typhoid fever is more common, such as South Asia or Latin America, have seen a higher increase in gallbladder cancer.^14^ Gallstones are an important risk factor owing to their high correlation with GBC. Studies have found high rates of gallbladder cancer incidence and gallstone prevalence in Pima Indian females, East Indian females.^14,15^

Historically, studies have aggregated the AsA population when investigating cancer diagnoses, treatments, and outcomes. However, an increasing body of evidence demonstrates that this heterogeneous group has numerous differences in cancer statistics when disaggregated in other cancer types.^8–12^ This paper builds on this body of evidence by looking at cancer outcomes in the disaggregated AsA population for gallbladder cancer, a type of cancer with generally poor outcomes. The data demonstrated that the aggregated AsA population had the most extended OS at 15.1 months compared with other ethnic demographics. However, when disaggregated, analysis showed a difference between the shortest OS of 12.6 months in Koreans and the longest of 19.1 months in Filipinos. To our knowledge, this is the first paper to look at gallbladder cancer in a disaggregated AsA population.

Previous studies have demonstrated the importance of socioeconomic barriers to access to care and financial status as predictive factors for survival with cancer.^16^ This study found that the AsA patient population tended to have the highest proportion of patients within the highest income bracket. While there is still generous heterogeneity in income between people within each ethnic grouping, these data may suggest that economic status is only part of the equation regarding patient outcomes. Specifically, with hepatobiliary cancers, increased travel distance is associated with poor survival.^17^ However, this analysis found that Filipino patients traveled the furthest median distance for treatment but had the highest OS.

High-quality care for GBC includes surgical resection and adjuvant therapies if feasible. For T1a disease, management is limited to cholecystectomy. Still, for more advanced tumors, surgical management extends to hepatic resection, including the gallbladder fossa, portal and retroperitoneal lymphadenectomy, and resection of the biliary ductal margin as indicated to achieve an R0 resection.^18,19^ While the NDCB is limited in distinguishing the extent of surgery patients received, previous literature has demonstrated the benefit of receiving care at high-volume centers.^20^ This paper’s data also shows that the Filipino population had the highest rate of surgical resection (67%) compared with other AsA groups. The Chinese and Korean populations had the lowest rates (55% and 60% respectively). This corresponded with lower OS in these groups.

It is not yet clear what role adjuvant chemotherapy plays in the treatment of gallbladder cancer. Two meta-analyses showed a nonsignificant survival benefit with adjuvant therapy and a significant survival benefit for R1 and node-positive disease.^21,22^ Additionally, the BILCAP phase III trial showed an overall survival benefit for 223 biliary tract cancers, which included 79 cases of GBC in a per-protocol analysis but did not show a significant survival benefit to treat.^23^ Overall, the current NCCN guidelines recommend adjuvant chemotherapy as part of the treatment algorithm for patients with GBC.^24^ This paper demonstrated that the Korean population had the highest rate of adjuvant chemotherapy (20%), whereas the Filipino group had the lowest rate (7%).

Patients who had received multimodality therapy were expected to have the highest OS in the population, but interestingly, it was found that Filipino patients had the most increased OS even while having a lower rate of treatment in academic/research centers, further travel distance, and no apparent differences in financial status. These findings suggest that socioeconomic factors and access to care are only part of the answer, which differs from studies that looked at pancreatic cancer in a disaggregated AsA population.^10^ Instead, we hypothesize that genetic and epigenetic influence likely drives patient outcomes. Tumor biology ultimately mediates how patients respond to therapy but is challenging to capture in retrospective database studies. There is limited data on the impact of ethnic and genetic variability on patients with gallbladder cancer. However, there is growing literature surrounding the integral role that epigenetic and genetic factors play in cancer etiology and treatment targets.

Epigenetics refer to modifications in gene function and expression without alteration to DNA sequence. There are several mechanisms in which epigenetics affect gene expression, such methylation, acetylation, or phosphorylation of histones, among many others.^25^ There are well-known examples of cancers associated with epigenetic changes in certain genes, such as hypermethylation of BRCA1 in breast cancer and hMLH1 in colon cancer.^26^ One comprehensive review by Feinberg et al.^27^ further advances a theory of epigenetic “landscapes” and its role in cancer etiology and potentially targeted therapies, such as tyrosine-kinase inhibitors. Furthermore, there is growing literature around the role of epigenetics in prognosis. Nagaraju et al.^28^ review the association of degrees of methylation and hepatocellular carcinoma (HCC) etiology, metastatic transformation, and survival outcomes in HCC patients. Specifically, hypermethylated CGI methylator phenotypes were associated with poor survival in HCC.^29,30^

While epigenetics in the propagation of cancer is well studied, the literature regarding differences in epigenetics between different ethnicities is still growing. There is a known racial disparity in breast cancer outcomes between African American women and Caucasian American women. Whereas robust literature surrounds the socioeconomic roots of these health disparities,^16^ epigenetic differences between races also is noted. One review discusses several genes that are differentially methylated in breast cancer between AA and CA women, e.g., methylation of *CDH13*, which is associated with triple negative breast cancers.^31–33^ Another systematic review of genetic and epigenetic differences in prostate cancer between African American patients and European American patients discussed differential methylation of multiple different gene promoters associated with prostate cancer.^34^ However, the clinical utility of these promoters are not certain.

There is sparse literature on the genetic and epigenetic profiles of Asian patients compared with Western patients. One study did show several genetic differences in prostate cancer tumors between Asian and Western patients,^35^ although they did not discuss epigenetic differences between races. Literature on epigenetic differences between races in regards to gallbladder cancer thus far has not been studied.

Further evidence of differential tumor biology based on race can be seen in gastric cancer. Gastric cancer has disproportionally affected Asian populations compared with Western populations. One study of SEER data showed that after adjusting for age, gender, tumor site, tumor grade, number of positive and total examined lymph nodes, and stage, Asian ethnicity was still a favorable prognostic factor for improved median and 5-year survival.^36^ A single-center study in the 1990s showed that the clinical behavior of patients with gastric cancer for patients of Asian descent was markedly different than the general population, which was also demonstrated in a recent NCDB analysis.^37,38^ Studies of the immune microenvironment for patients with gastric cancer have begun to suggest that specific tumor-infiltrating lymphocytes (TIL) are associated with differential survival. One study found that patients in the United States with increased survival had TIL profiles that were similar to those from Asian ancestry, which supports the notion that tumor biology is paramount.^39^ Similar studies are needed in gallbladder cancer and other hepatobiliary malignancies to characterize better genetic and epigenetic differences that mediate variation in survival.

A recent review also showed significant variability between tumor biology (among other factors, such as age at diagnosis, stage at diagnosis, tumor biology, treatment, and survival rates) of breast cancer between disaggregated subgroups of the AsA population.^9^ When controlled for socioeconomic differences, one investigation found that among AsA subgroups, Japanese women had a relatively high rate of hormone receptor-positive (HR+) disease and lower frequency of HER2+ and triple-negative disease compared with Korean, Filipina, and Vietnamese women.^40^ This growing body of literature demonstrating differences in tumor biology and pathology for other cancers based on disaggregated AsA subgroups independent of socioeconomic factors advances the notion of the important role genetic and epigenetic factors have on cancer outcomes.

Our study has limitations. The NCDB is a national retrospective database and is associated with limitations. Help is needed to understand specific patient tumor biology, surgical management, or specific chemotherapy regimens for individual patients in the dataset. Additionally, while this study attempted to control patient factors, it could not capture relevant cultural differences amongst patients, which are likely critical to explain differences in treatment utilization. Future institutional studies are needed to understand these populations’ patient decision-making better.

## Conclusions

This study identified differences in overall survival for patients with gallbladder cancer with a disaggregated AsA study design. Factors, such as socioeconomic barriers, cultural differences, and genetic and epigenetic causes, may explain these differences. This investigation highlights the importance of conducting research in a more disaggregated fashion to reduce disparities in access to care for patients of all ethnic backgrounds.

## Supplementary Information

Below is the link to the electronic supplementary material.

Supplementary file1 (DOCX 16 kb)
