# Supplementary material for: Unmasking Disparities in Gallbladder Cancer Outcomes in the Disaggregated Asian American Population
Source: Ann Surg Oncol. 2024 Sep 11;31(13):8699–711. doi: 10.1245/s10434-024-16168-x (PMC11549147; doi:10.1245/s10434-024-16168-x)
Supplement: Supplementary file 1 — Supplementary file1 (DOCX 16 kb) [file 10434_2024_16168_MOESM1_ESM.docx]

**Table S1: Distribution of Patients from Individual AsA Groups**

|  | **N** | **Percent** |
| --- | --- | --- |
| Chinese | 204 | 15.5 |
| Japanese | 82 | 6.2 |
| Filipino | 147 | 11.2 |
| Korean | 128 | 9.7 |
| Vietnamese | 98 | 7.4 |
| Laotian | 25 | 1.9 |
| Hmong | 17 | 1.3 |
| Kampuchean | 21 | 1.6 |
| Thai | 10 | 0.8 |
| Indian | 270 | 20.5 |
| Other Asian, including Asian, NOS and Oriental, NOS | 315 | 23.9 |
